# Influence of extender type, freezing temperature, and sperm concentration on the cryosurvival of Santa Inês ram spermatozoa

**DOI:** 10.1007/s11250-026-05435-6

**Published:** 2026-11-11

**Authors:** Fátima Révia Granja Lima, Paula Renata Cortat, Robson Mateus Freitas Silveira, Mirela Balistrieri, Renato Mesquita Peixoto, Ana Glaudia Catunda, Ana Claudia Nascimento Campos, Airtor Alencar Araújo

**Affiliations:** 1 Department of Animal Science, Center for Agricultural and Biological Sciences, State University Vale do Acaraú (UVA), Sobral, Ceará Brazil; 2 Department of Animal Science, “Luiz de Queiroz” College of Agriculture (ESALQ), University of São Paulo (USP), Piracicaba, São Paulo, Brazil; 3 Center for Education, Sciences and Technology of the Inhamuns Region, State University of Ceará (UECE), Tauá, Ceará Brazil; 4 Department of Animal Science, Center for Agricultural Sciences, Federal University of Ceará (UFC), Fortaleza, Ceará Brazil; 5 Federal Institute of Education, Science and Technology of Ceará (IFCE), Umirim Campus, Umirim, Ceará Brazil; 6 School of Veterinary Medicine, State University of Ceará (UECE), Fortaleza, Ceará Brazil

**Keywords:** Cryopreservation, CASA, Ovine semen, Sperm motility, Reproductive biotechnology

## Abstract

This study evaluated the effects of three extenders (Tris, HEPES, and skim milk), three freezing temperatures (-79, -90, and − 120 °C), and three final sperm concentrations (600, 800, and 1000 × 10⁶ spermatozoa/mL) on the post-thaw quality of Santa Inês ram semen. Semen was collected weekly from five rams during six consecutive weeks, pooled, and subjected to a 3 × 3 × 3 factorial arrangement. Samples were cryopreserved using a programmable freezing system (TK3000^®^) and stored in liquid nitrogen. After thawing, sperm quality was assessed by computer-assisted sperm analysis (CASA) and sperm morphology evaluation. Data were analyzed using mixed models, considering extender, freezing temperature, sperm concentration, and their interactions as fixed effects and collection week as a random effect. Tris consistently resulted in greater (*P* < 0.05) total motility, progressive motility, and percentage of rapid spermatozoa compared with HEPES and skim milk extenders. Semen frozen at 600 × 10⁶ spermatozoa/mL showed superior post-thaw motility characteristics relative to higher sperm concentrations. Freezing temperature affected sperm kinetics, particularly in skim milk and HEPES extenders, whereas Tris maintained stable performance across the evaluated temperatures. Total morphological abnormalities were minimally influenced by the treatments. Overall, under the experimental conditions evaluated, the combination of Tris extender, a freezing temperature of -79 °C, and a sperm concentration of 600 × 10⁶ spermatozoa/mL provided the most favorable post-thaw sperm quality among the treatments tested. However, progressive motility remained relatively low, indicating that further optimization and fertility validation are required before this combination can be recommended for practical application.

## Introduction

Artificial insemination is an important reproductive biotechnology for accelerating genetic improvement, disseminating superior germplasm, and preserving valuable genetic resources in livestock populations (Drechmer et al. 2024). In sheep production systems, the use of frozen semen allows long-term storage of genetic material and facilitates the exchange of superior sires across geographical regions. However, the widespread adoption of frozen-thawed ram semen remains limited because cryopreservation induces substantial structural and functional damage to spermatozoa, resulting in reduced fertility compared with fresh or chilled semen (Denk et al. 2026).

Ram spermatozoa are particularly susceptible to cryoinjury due to the unique lipid composition of their plasma membrane, which contains relatively low cholesterol concentrations and high proportions of polyunsaturated fatty acids (Salamon and Maxwell 2000; White 1993). During cooling, freezing, and thawing, sperm cells are exposed to osmotic stress, membrane phase transitions, oxidative damage, and intracellular ice crystal formation, all of which can impair sperm motility, membrane integrity, and fertilizing capacity (Parks and Lynch 1992; Watson 2000; Purdy et al. 2022). Consequently, optimization of cryopreservation protocols remains a major challenge for improving the efficiency of ovine artificial insemination programs.

Importantly, the response of ram spermatozoa to cryopreservation is not uniform across breeds. Differences among breeds have been reported in sperm quality and in the extent of cryodamage following the freeze-thaw process, suggesting that breed-specific characteristics may influence sperm cryotolerance (Barbas et al. 2023; Vozaf et al. 2022). Such variability is particularly relevant when cryopreservation protocols developed for one breed are applied to another, as the optimal combination of cryoprotective conditions may not be universally transferable. Therefore, breed-specific investigations are needed to establish cryopreservation protocols adapted to the biological characteristics of individual sheep populations.

Among the factors influencing cryosurvival, extender composition, sperm concentration, and freezing temperature play central roles in determining post-thaw sperm quality. Extenders provide buffering capacity, osmotic protection, energy substrates, and membrane stabilizers that help maintain sperm functionality throughout the cryopreservation process. Similarly, sperm concentration can influence the distribution of cryoprotective agents and the interaction among sperm cells during freezing, whereas freezing temperature directly affects the rate of water removal and ice crystal formation within the cell (Molinia et al. 1996; D’Alessandro et al. 2001; Gil et al. 2003). Although these factors have been investigated individually in ram semen, studies simultaneously evaluating their combined effects within the same experimental framework are scarce. Comprehensive factorial comparisons involving extender type, sperm concentration, and freezing temperature remain limited, making it difficult to determine whether the effects of one factor depend on the levels of the others.

The Santa Inês breed is the most important hair sheep breed in Brazil and plays a strategic role in meat production systems under tropical and subtropical conditions (Castro et al. 2021). Previous research has evaluated different sperm concentrations for cryopreservation of Santa Inês ram semen (Bittencourt et al. 2018; Menezes et al. 2020), and other studies have investigated alternative extenders and freezing conditions. However, a systematic comparison of these factors within a single experimental design is still lacking. This is relevant because Santa Inês rams are exposed to production conditions distinct from those of many temperate sheep breeds, and protocols developed under other genetic and environmental contexts may not necessarily provide optimal sperm cryosurvival in this breed.

Post-thaw sperm motility and morphology are widely used indicators of cryopreservation success because they provide complementary information on sperm function and structural integrity. Total and progressive motility reflect the ability of surviving spermatozoa to maintain movement and forward progression after thawing, whereas the proportion of rapid spermatozoa and kinematic parameters such as average path velocity (VAP) provide additional information on the characteristics of sperm movement that may be masked by conventional motility estimates (Ashrafi et al. 2011). Computer-assisted sperm analysis (CASA) enables objective quantification of these parameters and has been widely applied to evaluate the effects of cryopreservation and freezing conditions on ram sperm motility and kinematics (e.g., Yániz et al. 2005). Morphological abnormalities, in turn, provide an additional measure of structural damage associated with the freeze-thaw process. While these conventional metrics provide a solid baseline for cryosurvival, the development of functional assays, such as the ability of spermatozoa to bind to oviductal epithelial cells, is emerging as a valuable advanced strategy for fertility assessment, highlighting the complexity of sperm-female tract interactions (Cortat et al. 2026). Thus, evaluating structural and kinematic parameters together, while keeping functional competence in perspective, provides a broader assessment of sperm cryosurvival than total motility alone.

Therefore, we hypothesized that extender composition, freezing temperature, and sperm concentration interact to influence the cryosurvival of Santa Inês ram spermatozoa. The objective of this study was to evaluate the effects of three semen extenders (Tris, HEPES, and skim milk), three freezing temperatures (-79, -90, and − 120 °C), and three final sperm concentrations (600, 800, and 1000 × 10⁶ spermatozoa/mL) on post-thaw sperm motility and morphology of Santa Inês rams cryopreserved using a programmable freezing system.

## Materials and methods

### Experimental site

The experiment was conducted at the Laboratory of Andrology, Semen Technology, and Artificial Insemination (LATSIA) of Embrapa Goats and Sheep, located in Sobral, Ceará, Brazil (3°42′ S, 40°21′ W; 83 m above sea level). According to the Köppen climate classification, the region is characterized as a hot semi-arid climate (BSh) (Alvares et al. 2013). During May, the mean air temperature and relative humidity were approximately 26 °C and 60%, respectively.

### Experimental animals and ethical approval

Five sexually mature Santa Inês rams, aged between 24 and 48 months, were selected based on breeding soundness examinations conducted according to the guidelines described by Henry and Neves (1998). Throughout the experimental period, animals were housed in individual masonry pens under intensive management conditions.

The rams were fed chopped elephant grass (*Pennisetum purpureum*) ad libitum and received 500 g/day of a commercial concentrate (Ovino Top^®^, Integralmix, Brazil). Mineral salt and fresh water were provided *ad libitum* throughout the study period.

All experimental procedures involving animals were conducted in accordance with the ethical principles for animal experimentation established by the Brazilian National Council for the Control of Animal Experimentation (CONCEA) and complied with the provisions of Federal Law No. 11,794 of October 8, 2008 (Arouca Law), which regulates the scientific use of animals in Brazil. The study was carried out following institutional guidelines for animal welfare and good experimental practices.

### Experimental design

**Fig. 1:**
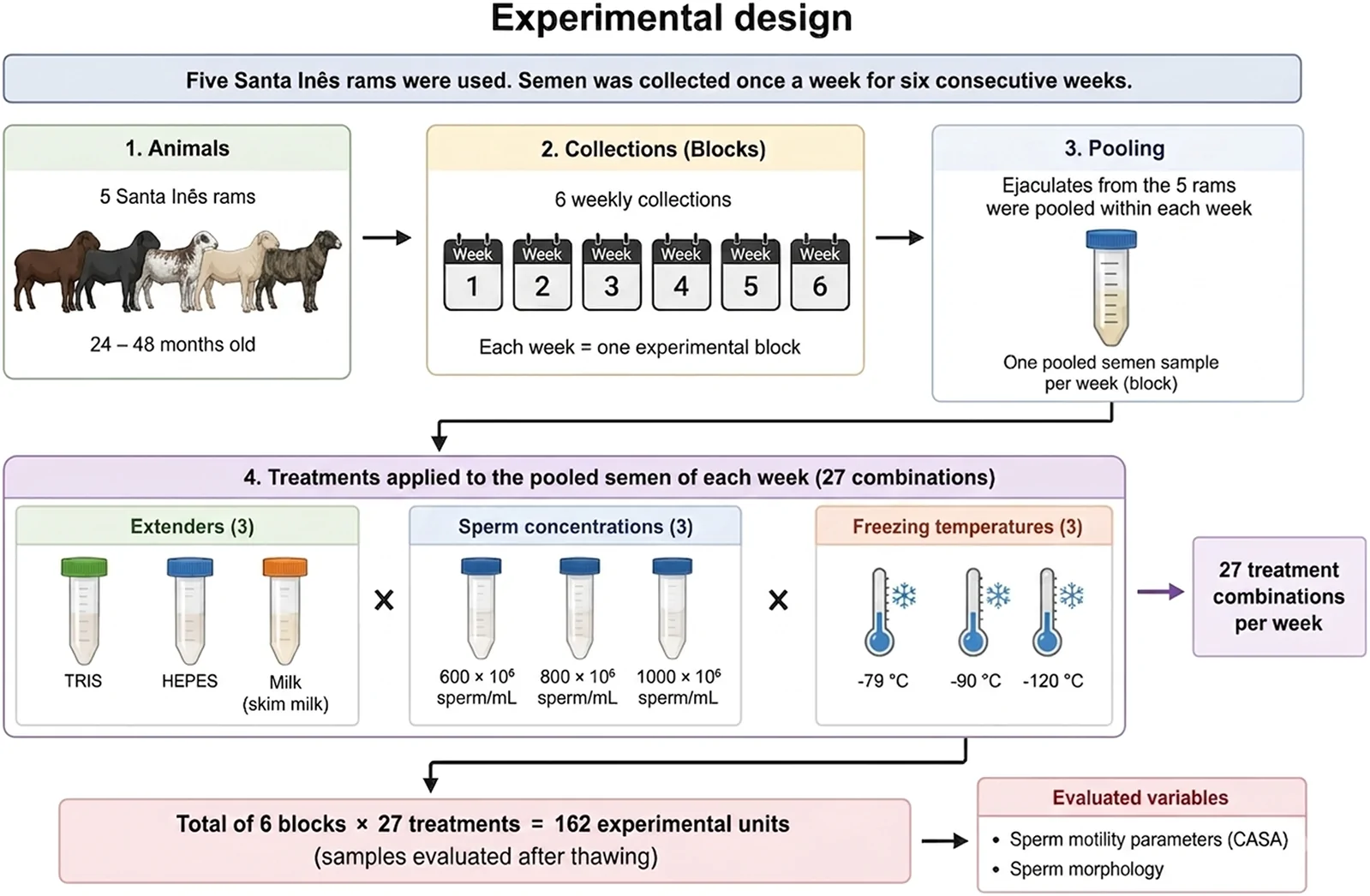
Schematic representation of experimental design. Semen was collected weekly from five Santa Inês rams during six consecutive weeks. At each collection, ejaculates were pooled to minimize individual animal variation, generating one semen pool per week (experimental block). Each pool was distributed among three extenders (Tris, HEPES, and skim milk), three final sperm concentrations (600, 800, and 1000 × 10⁶ spermatozoa/mL), and three freezing temperatures (-79, -90, and − 120 °C), resulting in 27 treatment combinations per block. A total of six blocks were evaluated, generating 162 experimental units. Post-thaw sperm quality was assessed by CASA and sperm morphology evaluation

The experiment was conducted over six consecutive weekly semen collections. At each collection, ejaculates obtained from the five Santa Inês rams were pooled to minimize individual animal variation and to provide sufficient semen volume for all treatments. Each weekly semen pool constituted one experimental block (Fig. 1).

The pooled semen was distributed among three extenders (Tris, HEPES, and skim milk), three final sperm concentrations (600, 800, and 1000 × 10⁶ spermatozoa/mL), and three freezing temperatures (-79, -90, and − 120 °C), resulting in 27 treatment combinations per collection. A total of six experimental blocks (weeks) were evaluated throughout the study, generating 162 experimental units (27 treatment combinations × 6 collection weeks).

### Extenders

Three semen extenders were prepared and evaluated in the present study. The Tris-based extender consisted of 76.5 mL of a stock solution containing 3.605 g Tris (hydroxymethyl)aminomethane, 1.488 g citric acid, and 2.024 g fructose dissolved in 100 mL of double-distilled water, supplemented with 20 mL chicken egg yolk, 3.5 mL glycerol, 100 IU penicillin, and 50 mg streptomycin, according to Evans and Maxwell (1987).

The skim milk-based extender consisted of 91.5 mL of a stock solution prepared with 10 g skim milk powder (Molico^®^, Nestlé, Brazil) and 0.194 g glucose dissolved in 100 mL distilled water, heated in a water bath for 10 min. After cooling to room temperature, the solution was supplemented with 5.0 mL chicken egg yolk, 3.5 mL glycerol, 100 IU penicillin, and 50 mg streptomycin, as described by Colas (1980).

The HEPES-based extender consisted of 76.5 mL of a stock solution containing 2.4 g N-(2-hydroxyethyl)piperazine-N′-(2-ethanesulfonic acid) (HEPES), 429 mg sodium bicarbonate, and 2.0 g glucose dissolved in 100 mL of double-distilled water, supplemented with 20 mL chicken egg yolk, 3.5 mL glycerol, 100 IU penicillin, and 50 mg streptomycin, according to Araújo (2000).

### Semen collection and processing

Semen was collected from five Santa Inês rams once weekly for six consecutive weeks using an artificial vagina maintained at 42 °C. Immediately after collection, ejaculates from all rams were pooled to minimize individual animal variation and to obtain sufficient semen volume for all cryopreservation treatments.

The pooled semen was maintained in a water bath at 32 °C and evaluated for volume, sperm motility, vigor, and sperm concentration. Sperm concentration was determined using a spectrophotometer. Subsequently, the semen pool was divided into nine aliquots corresponding to the three extenders (Tris, HEPES, and skim milk) and three final sperm concentrations (600, 800, and 1000 × 10⁶ spermatozoa/mL).

For each extender, semen was packaged into 0.25-mL plastic straws, generating three straws per sperm concentration. This procedure resulted in a total of 27 straws per collection (3 extenders × 3 sperm concentrations × 3 freezing temperatures).

Cryopreservation was performed using a programmable freezer (TK3000^®^, TK Tecnologia em Congelação Ltda., Brazil) with the P3S1 freezing program developed for ovine semen. Samples were cooled to 5 °C over a period of 76 min, followed by an equilibration period of 20 min. Freezing then proceeded for an additional 30 min until the final temperature of -120 °C was reached, characterizing a slow-freezing protocol.

To evaluate the effect of freezing temperature, straws were removed from the programmable freezer and transferred to liquid nitrogen (-196 °C) when temperatures of -79 °C, -90 °C, or -120 °C were reached. Consequently, each treatment combination was cryopreserved at one of the three predetermined freezing temperatures before long-term storage in liquid nitrogen.

### Post-thaw sperm evaluation

All semen samples remained stored in liquid nitrogen for 30 days before evaluation. Straws were thawed in a water bath at 37 °C for 20 s and subsequently diluted in a sodium citrate-glucose solution containing 2.37 g sodium citrate and 0.80 g glucose dissolved in 100 mL distilled water (Evans and Maxwell 1987) to obtain a final concentration of approximately 50 × 10⁶ spermatozoa/mL.

Post-thaw sperm motility was assessed using a CASA (Sperm Class Analyzer^®^, SCA version 3.2.0, Microptic S.L., Barcelona, Spain) coupled to a phase-contrast microscope equipped with a digital video camera. After 2 min of incubation at 37 °C, a 10-µL aliquot of each sample was loaded into a pre-warmed Makler counting chamber (Sefi Medical Instruments, Israel) and evaluated in five microscopic fields.

The CASA variables analyzed included total motility (%), progressive motility (%), percentage of rapid spermatozoa (%), and VAP (µm/s).

### Sperm morphology

Sperm morphological abnormalities were evaluated using bromophenol blue-stained smears according to the technique described by Medeiros et al. (2006). Smears were prepared after thawing semen, and a total of 200 spermatozoa per sample were examined under oil immersion at 1000× magnification using light microscopy. Sperm cells were classified according to the system proposed by Blom (1973), including normal spermatozoa and abnormalities affecting the head, midpiece, and tail, as well as proximal and distal cytoplasmic droplets. Total morphological abnormalities (TMA) were calculated as the percentage of spermatozoa presenting any morphological defect.

### Statistical analysis

Data were analyzed using a randomized complete block design, with collection week considered as a random blocking factor. The treatments were arranged in a 3 × 3 × 3 factorial arrangements, comprising three factors (extenders, sperm concentrations, and three freezing temperatures), with three levels for each factor.

Sperm motility and morphology variables were analyzed using linear mixed models implemented with the PROC MIXED of SAS (SAS Institute Inc., Cary, NC, USA). Semen extender, sperm concentration, freezing temperature, and their interactions were included as fixed effects, whereas collection week was included as a random effect to account for variation among semen collections. For interaction effects, the three-way interaction among semen extender, sperm concentration, and freezing temperature was tested first. Two-way interactions were considered and presented only when the corresponding three-way interaction was not significant. Thus, when a significant two-way interaction was identified in the absence of a significant three-way interaction, the effect was interpreted across the levels of the third factor.

When significant effects were detected, least-squares means were compared using Tukey’s adjustment for multiple comparisons. Residuals were examined for normality and homogeneity of variance through graphical inspection and diagnostic tests. Results are presented as least-squares means ± standard error of the mean (SEM), and differences were considered significant at *P* < 0.05.

## Results

Significant interactions between semen extender and freezing temperature were observed for total motility, progressive motility, the percentage of rapid spermatozoa, and average path velocity (*P* < 0.05). The interaction between semen extender and sperm concentration significantly affected total motility, progressive motility, and the percentage of rapid spermatozoa (*P* < 0.05), but not average path velocity (*P* > 0.05). A significant triple interaction among semen extender, freezing temperature, and sperm concentration was observed for total morphological abnormalities (*P* < 0.05). These interactions are further explored in detail below.

### Effect of extender and freezing temperature on sperm motility parameters

The effects of extender and freezing temperature on post-thaw sperm motility parameters are presented in Table 1. Total motility was consistently greater in semen cryopreserved with Tris compared with skim milk at all freezing temperatures (*P* < 0.05), whereas HEPES yielded intermediate values. Within extenders, total motility decreased at -90 °C and − 120 °C compared with − 79 °C in skim milk and HEPES, whereas no differences among freezing temperatures were observed for Tris (*P* > 0.05).

For progressive motility, a significant extender × freezing temperature interaction was observed. In skim milk, progressive motility was greater at -79 °C than at -90 °C and − 120 °C (*P* < 0.05). In HEPES, progressive motility was greater at -120 °C than at -90 °C (*P* < 0.05), whereas no differences among freezing temperatures were detected in Tris. Regardless of freezing temperature, Tris resulted in greater progressive motility than the other extenders (*P* < 0.05).

A similar pattern was observed for the percentage of rapid spermatozoa. Semen frozen in skim milk showed greater values at -79 °C than at the other temperatures (*P* < 0.05). In HEPES, the percentage of rapid spermatozoa was greater at -120 °C than at -90 °C (*P* < 0.05), whereas no effect of freezing temperature was detected in Tris. At all freezing temperatures, Tris produced greater percentages of rapid spermatozoa than HEPES and skim milk (*P* < 0.05).

For VAP, semen cryopreserved in skim milk showed greater values at -79 °C than at -90 °C and − 120 °C (*P* < 0.05). No differences among freezing temperatures were detected for HEPES or Tris. At -90 °C and − 120 °C, both HEPES and Tris exhibited greater VAP values than skim milk (*P* < 0.05), whereas no differences among extenders were observed at -79 °C.

**Table 1:** Effect of extender and freezing temperature on post-thaw sperm motility parameters of Santa Inês rams

Variable	Freezing temperature (°C)	Milk	HEPES	Tris
Total motility (%)				
	-79	40.8 ± 4.3 ^aB^	52.4 ± 4.6 ^aB^	66.8 ± 4.8 ^aA^
	-90	30.8 ± 5.0 ^aA^	44.7 ± 5.0 ^bB^	62.7 ± 4.2 ^aA^
	-120	33.8 ± 5.3 ^aB^	56.0 ± 4.4 ^aA^	62.5 ± 4.3 ^aA^
Progressive motility (%)				
	-79	8.0 ± 1.3 ^aB^	9.6 ± 1.4 ^abB^	15.5 ± 1.7 ^aA^
	-90	4.8 ± 1.8 ^bC^	8.1 ± 1.2 ^bB^	13.2 ± 1.3 ^aA^
	-120	5.4 ± 1.2 ^bC^	11.2 ± 1.6 ^aB^	13.6 ± 1.3 ^aA^
Rapid sperm (%)				
	-79	9.1 ± 1.5 ^aB^	11.4 ± 2.0 ^abB^	22.5 ± 3.6 ^aA^
	-90	5.9 ± 2.5 ^bC^	9.6 ± 1.6 ^bB^	17.3 ± 2.1 ^aA^
	-120	5.5 ± 1.1 ^bC^	13.4 ± 2.3 ^aB^	16.9 ± 2.1 ^aA^
VAP (µm/s)	-79	102.6 ± 4.3 ^aA^	110.4 ± 3.5 ^aA^	111.4 ± 3.6 ^aA^
	-90	90.9 ± 5.2 ^bB^	106.1 ± 4.3 ^aA^	109.1 ± 2.8 ^aA^
	-120	78.2 ± 6.2 ^bB^	108.0 ± 4.1 ^aA^	99.6 ± 6.3 ^aA^

Different superscript letters in lowercase within a column indicate differences among freezing temperatures within the same extender. Different superscript letters in uppercase within a row indicate differences among extenders within the same freezing temperature (*P* < 0.05). Values are presented as mean ± SEM

### Effect of extender and sperm concentration on sperm motility parameters

The effects of extender and sperm concentration on post-thaw sperm motility parameters are presented in Table 2. Total motility was affected by sperm concentration within all extenders. In milk, total motility was greater at 600 × 10⁶ and 800 × 10⁶ sperm/mL than at 1000 × 10⁶ sperm/mL (*P* < 0.05). In HEPES and Tris, the 600 × 10⁶ sperm/mL concentration resulted in greater total motility than 1000 × 10⁶ sperm/mL, whereas values obtained at 800 × 10⁶ sperm/mL were intermediate and did not differ from the other concentrations (*P* > 0.05). Among extenders, Tris produced the greatest total motility at 600 × 10⁶ sperm/mL (*P* < 0.05).

Progressive motility was also influenced by sperm concentration. In milk and Tris, the 600 × 10⁶ sperm/mL concentration resulted in greater progressive motility than the higher concentrations tested (*P* < 0.05). In HEPES, progressive motility was greater at 600 × 10⁶ sperm/mL than at 1000 × 10⁶ sperm/mL, whereas the value obtained at 800 × 10⁶ sperm/mL did not differ from either concentration. Across extenders, Tris at 600 × 10⁶ sperm/mL exhibited the greatest progressive motility (*P* < 0.05).

For the percentage of rapid spermatozoa, the 600 × 10⁶ sperm/mL concentration yielded the highest values in all extenders. However, in milk and HEPES, values obtained at 600 × 10⁶ and 800 × 10⁶ sperm/mL did not differ (*P* > 0.05). In Tris, the percentage of rapid spermatozoa decreased as sperm concentration increased, with the greatest values observed at 600 × 10⁶ sperm/mL (*P* < 0.05).

No effect of sperm concentration was detected on VAP within any extender (*P* > 0.05). Although numerically greater VAP values were generally observed at 600 × 10⁶ sperm/mL, particularly in milk and HEPES, no differences among extenders were detected for this variable.

**Table 2:** Effect of extender and sperm concentration on post-thaw sperm motility parameters of Santa Inês rams

Variable	Sperm concentration (×10⁶ sperm/mL)	Skim milk	HEPES	Tris
Total motility (%)	600	44.0 ± 5.0 ^aC^	61.3 ± 4.1 ^aB^	79.0 ± 2.0 ^aA^
	800	36.0 ± 4.8 ^aC^	49.8 ± 5.0 ^abB^	63.7 ± 4.6 ^aA^
	1000	25.3 ± 4.1 ^bB^	42.0 ± 4.0 ^bA^	50.4 ± 3.2 ^bA^
Progressive motility (%)	600	9.5 ± 1.9 ^aB^	12.7 ± 1.6 ^aB^	18.4 ± 1.4 ^aA^
	800	5.9 ± 1.2 ^bC^	9.1 ± 1.4 ^abBC^	13.8 ± 1.3 ^bA^
	1000	2.9 ± 0.6 ^bC^	7.1 ± 0.8 ^bA^	10.4 ± 1.0 ^bA^
Rapid sperm (%)	600	10.5 ± 2.5 ^aB^	15.9 ± 2.5 ^aB^	26.5 ± 2.7 ^aA^
	800	6.5 ± 1.4 ^abB^	10.8 ± 1.8 ^abB^	18.5 ± 2.3 ^bA^
	1000	3.5 ± 0.8 ^bC^	7.7 ± 0.9 ^bB^	12.3 ± 1.3 ^bcA^
VAP (µm/s)	600	98.5 ± 6.0 ^aA^	111.5 ± 2.9 ^aA^	104.0 ± 6.4 ^aA^
	800	88.4 ± 5.7 ^aB^	106.3 ± 4.3 ^aA^	108.4 ± 3.7 ^aA^
	1000	84.8 ± 5.2 ^aB^	106.0 ± 4.6 ^aA^	107.8 ± 3.0 ^aA^

Values are presented as mean ± SEM. Different superscript letters in lowercase within a column indicate differences among sperm concentrations within the same extender. Different superscript letters in uppercase within a row indicate differences among extenders within the same sperm concentration (*P* < 0.05)

### Effect of extender, sperm concentration, and freezing temperature on total morphological abnormalities

The TMA were generally similar among extenders, freezing temperatures, and sperm concentrations (Table 3). Within the milk extender, greater TMA was observed at -90 °C than at -120 °C when semen was frozen at 800 × 10⁶ sperm/mL (*P* < 0.05). No differences were detected among treatments within HEPES (*P* > 0.05). In Tris, at 1000 × 10⁶ sperm/mL, TMA was lower at -79 °C than at -90 °C (*P* < 0.05). No additional treatment effects were observed.

**Table 3:** Effect of extender, sperm concentration, and freezing temperature on total morphological abnormalities (TMA, %) of frozen-thawed sperm from Santa Inês rams

Sperm concentration (×10⁶ sperm/mL)		Freezing temperature (°C)	Milk		HEPES	Tris
600	-79			21.9 ± 4.2	20.3 ± 2.9	22.8 ± 7.3
	-90			19.4 ± 4.0	18.5 ± 3.7	20.2 ± 5.1
	-120			19.2 ± 6.0	20.4 ± 5.1	18.5 ± 2.7
800	-79			18.7 ± 5.4^b^	17.6 ± 4.6	19.3 ± 2.0
	-90			22.1 ± 3.4^a^	20.0 ± 5.3	20.4 ± 5.2
	-120			17.6 ± 3.6^b^	19.6 ± 3.3	21.3 ± 5.0
1000	-79			18.7 ± 4.9	16.1 ± 4.1	17.9 ± 4.9^a^
	-90			16.6 ± 3.6	19.0 ± 5.0	22.4 ± 5.3^a^
	-120			19.0 ± 4.0	18.3 ± 3.5	18.8 ± 4.1^ab^

Values are presented as mean ± SEM. Different superscript letters in lowercase within the same extender and sperm concentration indicate differences among freezing temperatures (*P* < 0.05)

## Discussion

The present study demonstrated that post-thaw sperm quality in Santa Inês rams was influenced by both extender composition and sperm concentration. Overall, Tris resulted in superior sperm motility parameters compared with milk and HEPES extenders, particularly for progressive motility and the percentage of rapid motile sperm. In addition, the lowest sperm concentration evaluated (600 × 10⁶ sperm/mL) consistently yielded the best post-thaw motility results, whereas total morphological abnormalities were only minimally affected by the evaluated treatments. Despite these relative differences among treatments, progressive motility remained low across all experimental conditions, with the highest value reaching only 18.4%. Therefore, the observed treatment effects should be interpreted considering the overall low progressive motility observed after thawing.

The superior performance of the Tris extender observed in the present study agrees with previous reports demonstrating the effectiveness of Tris-based extenders for ram semen cryopreservation. Extender composition plays a fundamental role in protecting sperm cells against cryoinjuries, since buffering systems, cryoprotectants, and membrane-stabilizing components directly influence sperm survival during freezing and thawing. Similar results were reported by Carvalho et al. (2008), who observed superior post-thaw sperm quality in ram semen cryopreserved in Tris-based extenders compared with milk-based formulations. More recently, studies evaluating Tris-based extenders supplemented with antioxidants have reported improvements in post-thaw sperm motility, kinematic parameters, and membrane integrity, reinforcing the suitability of Tris as a cryopreservation medium for ram semen (Souza et al. 2019; Brair et al. 2024). Furthermore, contemporary reviews continue to identify Tris-based systems as among the most effective extenders for ovine semen cryopreservation due to their buffering capacity and ability to maintain sperm functionality after thawing (Purdy et al. 2022).

The beneficial effects of Tris may be associated with its zwitterionic buffering capacity and its ability to maintain membrane stability throughout the cryopreservation process. In addition, egg yolk components could contribute to sperm protection by reducing membrane destabilization during cooling and freezing. Since egg yolk was included in all formulations evaluated in this study, the superiority of Tris over milk and HEPES cannot be attributed solely to egg-yolk lipoproteins. Instead, it reflects the superior ability of Tris to prevent pH shifts and osmotic distress during freezing, whereas milk casein matrices lack equivalent buffering power and HEPES experiences temperature-dependent pH drops. Such mechanisms may partially explain the superior progressive motility and rapid sperm percentages observed in semen frozen with the Tris extender. Furthermore, Santa Inês rams may exhibit unique membrane lipid compositions compared with temperate wool breeds, which may render their sperm particularly dependent on the robust osmotic protection provided by Tris.

Freezing temperatures also affected sperm motility characteristics. In the milk extender, sperm frozen at -79 °C exhibited greater progressive motility, rapid sperm percentage, and VAP compared with lower freezing temperatures. In contrast, no significant differences among freezing temperatures were observed for most motility variables in the Tris extender, suggesting greater resistance of sperm cells to temperature variation during cryopreservation. According to Watson (2000), cryopreservation-induced damage results from complex interactions among osmotic stress, membrane phase transitions, and intracellular ice formation. Therefore, even small changes in cooling or freezing rates may substantially affect sperm survival. Similarly, Holt (2000), Salamon and Maxwell (1995), and Purdy et al. (2022) emphasized that optimal freezing conditions depend on the interaction between semen characteristics, extender composition, cooling rate, and cryopreservation protocol.

The interaction between extender and freezing temperature observed for progressive motility and rapid sperm percentage suggests that the response of sperm cells to freezing is not determined solely by temperature, but also by the protective capacity of the extender. Previous studies have reported conflicting results regarding the optimal freezing temperature for ram semen, which likely reflect differences in extender composition, glycerol concentration, freezing systems, and semen processing protocols (D’Alessandro et al. 2001; El-Alamy and Foote 2001). The superior performance of Tris at -79 °C observed in the present study indicates that this combination provided the most favorable conditions for preserving sperm motility under the experimental conditions evaluated. Although not directly tested here, other processing variables such as equilibration time and cooling rates are well-documented to modulate these responses by allowing adequate cryoprotectant permeation before freezing (Salamon and Maxwell 2000).

It is important to acknowledge that progressive motility remained low across treatments, even under the conditions that resulted in the greatest values. All extenders evaluated in the present study contained 3.5% glycerol. Previous studies have shown that glycerol concentration can influence post-thaw ram sperm quality, with concentrations ranging from approximately 4 to 7% frequently reported as providing effective cryoprotection, although the optimal concentration depends on extender composition and freezing conditions (Gil et al. 2003; Purdy et al. 2022). Therefore, the relatively low glycerol concentration used in the present study may have contributed to the low progressive motility observed after thawing. However, because glycerol concentration was not evaluated as an experimental factor, its specific contribution cannot be determined.

Sperm concentration exerted a consistent effect on post-thaw sperm motility. In general, semen frozen at 600 × 10⁶ sperm/mL exhibited greater total motility, progressive motility, and rapid sperm percentage than semen frozen at higher concentrations. These findings agree with D’Alessandro et al. (2001), who reported detrimental effects of increased sperm concentration on post-thaw sperm quality and fertility in sheep. Similarly, Ritar and Ball (1993) suggested that high sperm density may increase unfavorable interactions among sperm cells, seminal plasma components, and extender constituents, ultimately reducing post-thaw sperm function. Salamon and Maxwell (2000) also reported that reduced dilution rates and higher sperm concentrations may compromise cryosurvival and fertility of frozen-thawed ram semen.

The superior motility observed at 600 × 10⁶ sperm/mL may be associated with improved distribution of cryoprotective components around sperm cells and reduced cellular competition during freezing and thawing. Previous studies have demonstrated that excessive sperm concentrations can negatively affect membrane integrity, acrosomal preservation, and overall sperm functionality after cryopreservation (D’Alessandro et al. 2001; Molinia et al. 1996). Similarly, the detrimental effects of higher sperm concentrations on post-thaw sperm quality have been consistently reported in small ruminants (Salamon and Maxwell 2000; Purdy et al. 2022).

In contrast to motility parameters, total morphological abnormalities were only slightly affected by extender, sperm concentration, and freezing temperature. Most values remained within a relatively narrow range, indicating that sperm morphology was less sensitive to treatment effects than sperm motility. These findings agree with previous reports indicating that sperm motility is often more sensitive to cryopreservation-induced damage than conventional morphological evaluations (Salamon and Maxwell 1995; Watson 2000). Although morphology remains an important component of semen evaluation, the present results suggest that extender composition, freezing temperature, and sperm concentration exerted a greater influence on sperm kinetics than on sperm morphology.

A limitation of the present study is that sperm quality was evaluated exclusively through post-thaw motility and morphological assessments. In addition, the CASA evaluation included a limited set of sperm kinetic parameters. Additional functional analyses, such as plasma membrane integrity, acrosomal status, mitochondrial activity, oxidative stress markers, and fertility trials, could provide a more comprehensive understanding of the biological consequences of the cryopreservation protocols evaluated. Nevertheless, CASA-derived motility parameters remain among the most widely used and informative indicators of post-thaw sperm quality, particularly in ram semen cryopreservation studies (Mocé and Graham 2008; Purdy et al. 2022).

The CASA has become an important tool for identifying subtle differences in sperm kinematics following cryopreservation. Parameters such as progressive motility and velocity measurements are considered relevant indicators of sperm functionality and semen quality (Mocé and Graham 2008). More recently, Silva et al. (2026) reported differences in CASA-derived kinematic parameters between sires with contrasting fertility, reinforcing the biological relevance of sperm motion characteristics. Likewise, Silva et al. (2025) demonstrated that conventional semen quality parameters alone may not fully explain differences in reproductive performance, highlighting the importance of evaluating complementary sperm functional attributes. In the present study, the superiority of the Tris extender and the lower sperm concentration were consistently reflected in progressive motility and rapid sperm percentages, indicating that these variables were sensitive to differences among the cryopreservation conditions evaluated.

The present results reinforce the importance of evaluating multiple sperm quality parameters after cryopreservation. Conventional semen analyses, including motility and morphology, remain essential tools for assessing sperm quality and cryosurvival (Oliveira et al. 2014; Mocé and Graham 2008).

In conclusion, the combination of a Tris extender, a freezing temperature of -79 °C, and a final sperm concentration of 600 × 10⁶ sperm/mL provided the most favorable post-thaw sperm motility characteristics among the conditions evaluated in Santa Inês ram semen cryopreserved using the TK3000 system. Further studies evaluating protocol optimization and fertility outcomes following artificial insemination are warranted before this combination can be recommended for practical application.

## Data Availability

The data supporting the findings of this study are available from the corresponding author upon reasonable request.

## References

[CR37] Alvares CA, Stape JL, Sentelhas PC, de Moraes Gonçalves JL, Sparovek G (2013) Köppen’s climate classification map for Brazil. Meteorol Z 22:711–728. 10.1127/0941-2948/2013/0507

[CR1] Araújo AA (2000) Mise au point d´un dilueur de conservation en milieu liquide pour la semence ovine en vue de l´insémination artificielle [Tese de Doutorado em Ciências da Vida. Université François Rabelais de Tours]. Tours, França

[CR2] Ashrafi I, Kohram H, Naijian H, Bahreini M, Mirzakhani H (2011) Effect of controlled and uncontrolled cooling rate on motility parameters of cryopreserved ram spermatozoa. BMC Res Notes 4:547. 10.1186/1756-0500-4-547 PMC3349620 22185483

[CR3] Barbas JP, Pimenta J, Baptista MC et al (2023) Ram semen cryopreservation for Portuguese native breeds: Season and breed effects on semen quality variation. Animals 13:579. 10.3390/ani13040579 PMC9951670 36830367

[CR34] Bittencourt RF, Oba E, de Almeida Biscarde CE, Azevedo HC, Vasconcelos Bittencourt M, Oliveira de Menezes GF, da Silva Lima A, da Mata Fuchs K, Ribeiro Filho AL (2018) Dimethylacetamide and trehalose for ram semen cryopreservation. Cryobiol 85:1–6. 10.1016/j.cryobiol.2018.10.266 30391233

[CR5] Blom E (1973) The ultrastructure of some characteristic sperm defects and a proposal for a new classification of the bull spermiogram. Nordisk Veterinaermedicin 25:383–391 4768226

[CR6] Brair VL, Souza-Fabjan JMG, Brandão FZ et al (2024) The association of resveratrol and AFPI did not enhance the cryoresistance of ram sperm. Theriogenology 223:89–96 10.1590/1984-3143-AR2023-0159 PMC10878549 38384723

[CR7] Carvalho FP, Silva JFS, Souza GV, Quirino CR, Carvalho CSP (2008) Diferentes diluentes sobre a motilidade e integridade de membrana plasmática após o congelamento e descongelamento de sêmen ovino. Revista Brasileira de Saúde e Produção Anim 9(3):612–620

[CR33] Castro MSM, Vasconcelos AM, Santos VO, Lopes AKC, Farias MRS, Lima FRG, Rogério MCP, Ferreira J, Silveira RMF (2021) Thermoregulatory capacity of Santa Inês hair ewes of different genotypes associated with coat colors raised in a hot environment. J Appl Anim Welf Sci 24:400–410. 10.1080/10888705.2020.1819808 32951463

[CR8] Colas G (1980) Variations saisonnieres de la qualité du sperme chez le belier ile de France I. Etude de la morphologie cellulaire et de la motilité massale. Reprod Nutr Dev 20(6):1789–1799 7349512

[CR9] Cortat PR, de Souza CA, Bridi A, Silva e, Balistrieri LO, Schneberger M, Celeghini F, da Silveira EC, Carvalho JC, J. O., Sartori R (2026) Spermatozoa binding to oviduct cells: the influence of the endocrine milieu on the effectiveness of the binding test. Theriogenology 252:117761. 10.1016/j.theriogenology.2025.117761 41270603

[CR32] D’Alessandro AG, Martemucci G, Colonna MA, Bellitti A (2001) Post-thaw survival of ram spermatozoa and fertility after insemination as affected by prefreezing sperm concentration and extender composition. Theriogenol 55(5):1159–1170. 10.1016/S0093-691X(01)00474-5 11322242

[CR10] Denk B, Kırıkkulak M, Güngör Ş, Gülhan MF, İnanç ME, Avdatek F, Yeni D, Taşdemir U (2026) Enhancing post-thaw sperm quality in rams: quinic acid as a natural antioxidant. Front Veterinary Sci 12:1744161. 10.3389/fvets.2025.1744161 PMC12812912 41560853

[CR11] Drechmer J, Basílio LMS, Teixeira HCA, Ramos AF, Silva BDM (2024) Resynchronization of estrus in sheep using two or three artificial inseminations with frozen semen. Ciência Anim Brasileira 25:76929E. 10.1590/1809-6891v25e-76929E

[CR12] El-Alamy MA, Foote RH (2001) Freezability of spermatozoa from Finn and Dorset rams in multiple semen extenders. Anim Reprod Sci 65:245–254 10.1016/s0378-4320(00)00230-x 11267804

[CR13] Evans G, Maxwell WMC (1987) Salamon’s artificial insemination of sheep and goats. Butterworth

[CR14] Gil J, Lundeheim N, Söderquist L, Rodríguez-Martínez H (2003) Influence of extender, temperature, and addition of glycerol on post-thaw sperm parameters in ram semen. Theriogenology 59:1241–1255 10.1016/s0093-691x(02)01177-9 12527072

[CR15] Henry M, Neves JP (1998) Manual para Exame Andrológico e Avaliação de Sêmen Animal. Colégio Brasileiro de Reprodução Animal

[CR16] Holt WV (2000) Basic aspects of frozen storage of semen. Anim Reprod Sci 62:3–22 10.1016/s0378-4320(00)00152-4 10924818

[CR17] Medeiros AA, Araujo AA, Rodrigues LFS (2006) Utilização do azul de bromofenol conservado a 4°C e 29°C, como método de coloração vital para avaliação do espermatozóide ovino. Revista de Ciências Agrárias 46(1):287–297

[CR35] Menezes GFO de, Bittencourt RF, Cardoso F de L, Lents MP, Santos ES dos, Barreto RO, Jesus EO de, Valverde MM, Ribeiro Filho A de L (2020) Dimethylacetamide alone or in combination with glycerol can be used for cryopreservation of ovine semen. Anim Reprod 17(4):e20200036. 10.1590/1984-3143-AR2020-0036 PMC7995265 33791025

[CR18] Mocé E, Graham JK (2008) Sperm cryopreservation: evaluation of sperm quality. Theriogenology 69:423–436

[CR19] Molinia FC, Evans G, Maxwell WMC (1996) Fertility of ram spermatozoa pellet-frozen in zwitterion-buffered diluents. Reprod Nutr Dev 36:21–29 10.1051/rnd:19960102 8881589

[CR20] Oliveira LZ, Arruda RP, Andrade AFC, Celeghini ECC, Reeb PD, Martins JPN, Santos RM, Bezerra FSB, Henry M (2014) Assessment of field fertility and several in vitro sperm characteristics in bulls. Anim Reprod Sci 146:14–23

[CR21] Parks JE, Lynch DV (1992) Lipid composition and thermotropic phase behavior of boar, bull, stallion, and rooster sperm membranes. Cryobiology 29:255–266 10.1016/0011-2240(92)90024-v 1582232

[CR22] Purdy PH et al (2022) Cryopreservation techniques for ram sperm. Animals 12:1268

[CR23] Ritar AJ, Ball PD (1993) The effect of freeze-thawing of goat and sheep semen at a high density of spermatozoa on cell viability and fertility after insemination. Anim Reprod Sci 31:249–262

[CR24] Salamon S, Maxwell WMC (1995) Frozen storage of ram semen I. Processing, freezing, thawing and fertility after cervical insemination. Anim Reprod Sci 37:185–249

[CR25] Salamon S, Maxwell WMC (2000) Storage of ram semen. Anim Reprod Sci 62(1–3):77–111. 10.1016/S0378-4320(00)00155-X 10924821

[CR26] Silva MA, Cortat PR, Consentini CEC, Pinto SCC, Celeghini ECC, Carvalho JO, Melo LF, Wiltbank M, Sartori R (2025) Predicting sire fertility in artificial insemination of dairy cows by the ability of spermatozoa to bind to oviduct cell aggregates. J Dairy Sci 108(11):12722–12733. 10.3168/jds.2025-27057 40846065

[CR27] Silva MA da, Souza PRC de, Silva LO e, Consentini CEC, Madureira G, Monteiro PLJ, Sá Filho MF de, Celeghini ECC, Carvalho JO, Sartori Filho R. (2026) Contrasting parameters of higher vs. lower fertility Bos indicus sires: assessment of sperm binding to oviduct cells and adjustments in the timed artificial insemination protocol. Theriogenology 263:1–11. 10.1016/j.theriogenology.2026.117985 42167070

[CR28] Souza JMG, Brandão FZ et al (2019) Effect of different concentrations of L-carnitine in extender for semen cryopreservation in sheep. Cryobiology 89:104–109 10.1016/j.cryobiol.2019.05.009 31121151

[CR29] Vozaf J, Svoradová A, Baláži A et al (2022) The cryopreserved sperm traits of various ram breeds: Towards biodiversity conservation. Animals 12:1311. 10.3390/ani12101311 PMC9137843 35625160

[CR30] Watson PF (2000) The causes of reduced fertility with cryopreserved semen. Anim Reprod Sci 60–61:481–492 10.1016/s0378-4320(00)00099-3 10844218

[CR31] White IG (1993) Lipids and calcium uptake of sperm in relation to cold shock and preservation: a review. Reprod Fertility Dev 5(6):639–658. 10.1071/RD9930639 9627725

[CR36] Yániz J, Martí JI, Silvestre MA, Folch J, Santolaria P, Alabart JL, López-Gatius F (2005) Effects of solid storage of sheep spermatozoa at 15 °C on their survival and penetrating capacity. Theriogenology 64(8):1844–1851. 10.1016/j.theriogenology.2005.04.012 15904955

